# Supplementary figures and images for: Nelfinavir inhibition of Kaposi’s sarcoma-associated herpesvirus protein expression and capsid assembly
Source: Infect Agent Cancer. 2024 Mar 4;19:7. doi: 10.1186/s13027-024-00566-7 (PMC10913605; doi:10.1186/s13027-024-00566-7)

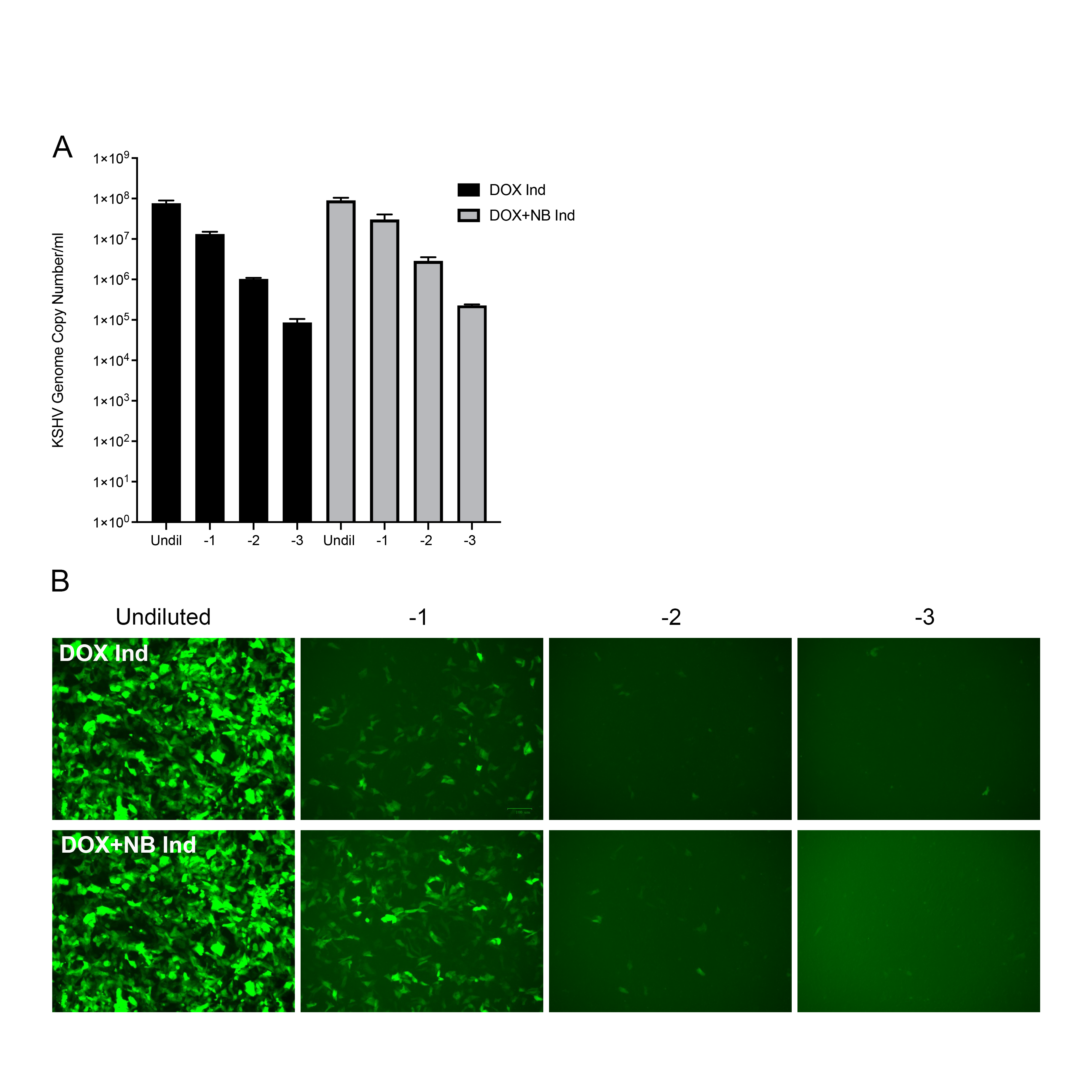

Supplement: Supplementary file 1 — Supplementary Material 1. Fig. S1. Titration of KSHV virus produced from 5r219 cells. 5r219 cells (1 x10^6^) were induced with doxycycline (DOX) or doxycycline plus 1 mM sodium butyrate (DOX+NB) and virus supernatants harvested, 72 h post-induction. The virus in the supernatants were concentrated by centrifugation and resuspended in PBS overnight at +4ºC. The virus titer was quantitated by qPCR assays (A) and infection of Vero cell monolayers (B). Undiluted (Undil) as well as serial ten-fold dilutions were assayed using both methods. [file 13027_2024_566_MOESM1_ESM.tif]

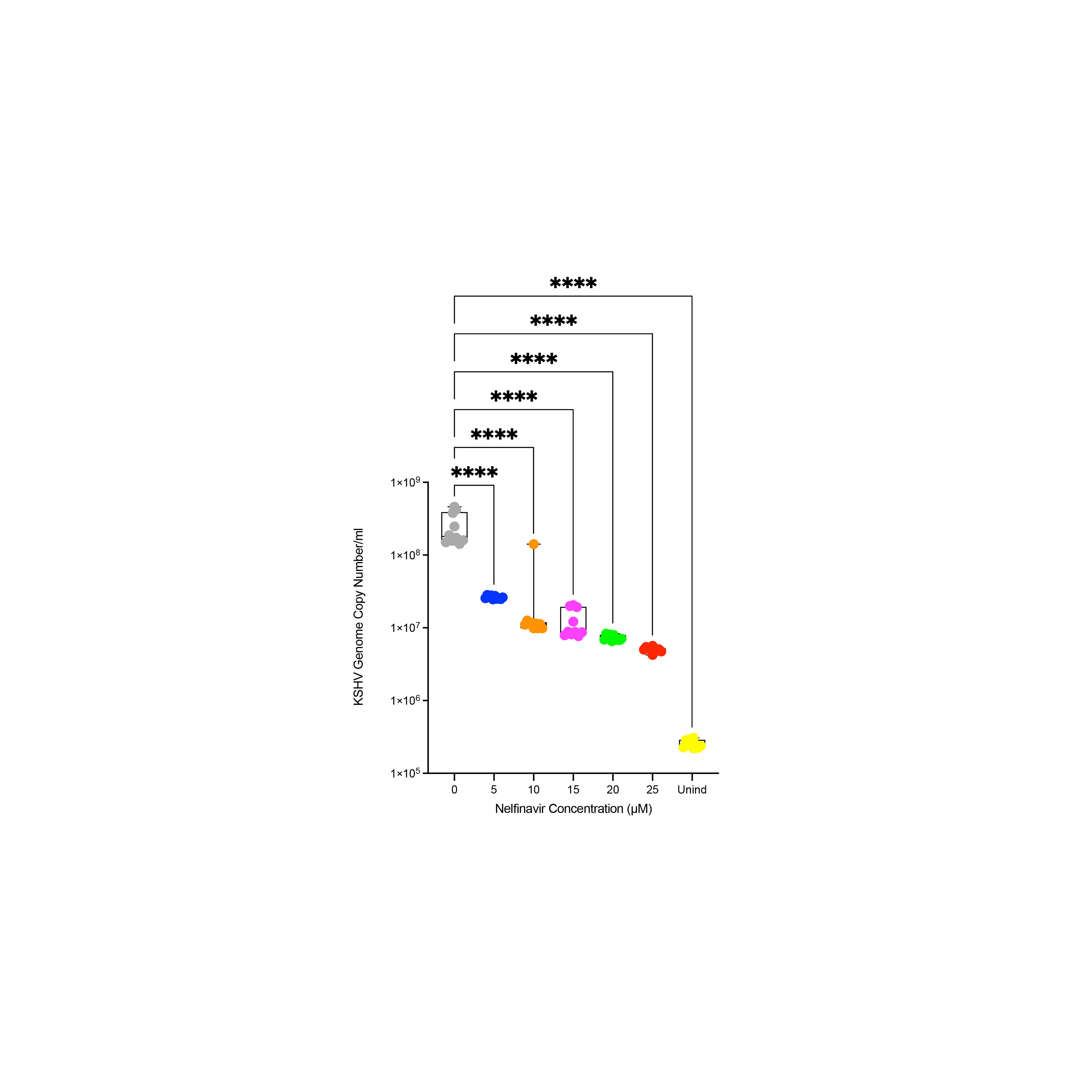

Supplement: Supplementary file 2 — Supplementary Material 2. Fig. S2. Statistical analysis of nelfinavir inhibition of KSHV genome copy number. One way ANOVA was used to calculate p values for data in Fig. 2A. For all comparisons we derived a p value of **** p<0.0001 which is statistically significant. All statistical analyses were performed with Prism V.9.0 Software (GraphPad Software). [file 13027_2024_566_MOESM2_ESM.tif]

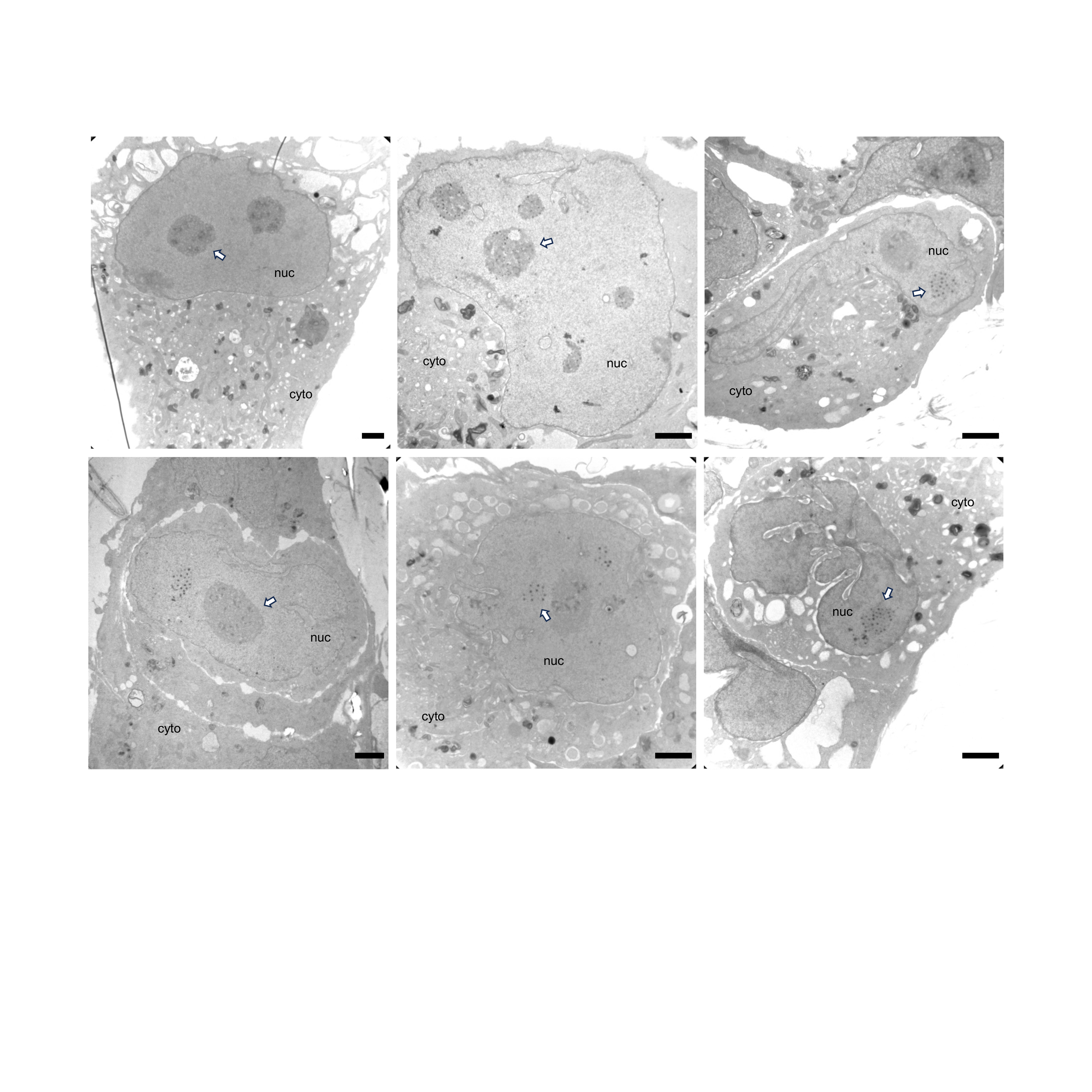

Supplement: Supplementary file 3 — Supplementary Material 3. Fig. S3. Ultrastructural analyses of nelfinavir treated 5r219 cells. 5r219 cells were incubated with 20 µM nelfinavir for 72 h following induction. Whole cells were imaged by TEM. The panels show the nuclei (nuc) and cytoplasmic (cyto) regions of these cells. Electron dense aggregates were evident in the nuclei (white arrowheads). No mature or enveloped virions were observed in the cytoplasm of the nelfinavir treated cells. Scale bar = 2000 micron. [file 13027_2024_566_MOESM3_ESM.tif]
