# Supplementary material for: Nelfinavir inhibition of Kaposi’s sarcoma-associated herpesvirus protein expression and capsid assembly
Source: Infect Agent Cancer. 2024 Mar 4;19:7. doi: 10.1186/s13027-024-00566-7 (PMC10913605; doi:10.1186/s13027-024-00566-7)
Supplement: Supplementary file 4 — Supplementary Material 4. Table S1. Antibodies used in the immuno-blot experiments presented here. [file 13027_2024_566_MOESM4_ESM.docx]

**Supplemental Table S1.** Antibodies used in the immuno-blot experiments presented here.

| Target Antigen | Host Species | Source |
| --- | --- | --- |
| RTA  (Replication and transcriptional activator) | Rabbit | Gary Hayward [1, 2] |
| MTA  (mRNA transcript accumulation) | Rabbit | Gary Hayward [1] |
| SSB  (single strand binding) | Rabbit | Gary Hayward [3] |
| vIRF1  (viral interferon regulatory factor 1) | Rabbit | Gary Hayward [4, 5] |
| vIL6  (viral interleukin 6) | Rabbit | Gary Hayward [6, 7] |
| K8  (KSHV Zebra homologue) | Rabbit | Gary Hayward [3] |
| ORF26  (Triplex protein 2) | Mouse | Novus Biologicals  (2F6B8) |
| CHOP  (C/EBP Homologous Protein) | Mouse | Cell Signaling  **(**L63F7) |
| GAPDH  (glyceraldehyde-3-phosphate dehydrogenase) | Rabbit | Invitrogen  (PA1-987) |

1. Malik P, Blackbourn DJ, Cheng MF, Hayward GS, Clements JB: **Functional co-operation between the Kaposi's sarcoma-associated herpesvirus ORF57 and ORF50 regulatory proteins**. *J Gen Virol* 2004, **85**(Pt 8):2155-2166.

2. Wang SE, Wu FY, Fujimuro M, Zong J, Hayward SD, Hayward GS: **Role of CCAAT/enhancer-binding protein alpha (C/EBPalpha) in activation of the Kaposi's sarcoma-associated herpesvirus (KSHV) lytic-cycle replication-associated protein (RAP) promoter in cooperation with the KSHV replication and transcription activator (RTA) and RAP**. *J Virol* 2003, **77**(1):600-623.

3. Wu FY, Ahn JH, Alcendor DJ, Jang WJ, Xiao J, Hayward SD, Hayward GS: **Origin-independent assembly of Kaposi's sarcoma-associated herpesvirus DNA replication compartments in transient cotransfection assays and association with the ORF-K8 protein and cellular PML**. *J Virol* 2001, **75**(3):1487-1506.

4. Vo MT, Choi CY, Choi YB: **The mitophagy receptor NIX induces vIRF-1 oligomerization and interaction with GABARAPL1 for the promotion of HHV-8 reactivation-induced mitophagy**. *PLoS Pathog* 2023, **19**(7):e1011548.

5. Ma Z, Jacobs SR, West JA, Stopford C, Zhang Z, Davis Z, Barber GN, Glaunsinger BA, Dittmer DP, Damania B: **Modulation of the cGAS-STING DNA sensing pathway by gammaherpesviruses**. *Proc Natl Acad Sci U S A* 2015, **112**(31):E4306-4315.

6. Yan Q, Zhou J, Wang Z, Ding X, Ma X, Li W, Jia X, Gao SJ, Lu C: **NAT10-dependent N(4)-acetylcytidine modification mediates PAN RNA stability, KSHV reactivation, and IFI16-related inflammasome activation**. *Nat Commun* 2023, **14**(1):6327.

7. Chen D, Nicholas J: **Promotion of Endoplasmic Reticulum-Associated Degradation of Procathepsin D by Human Herpesvirus 8-Encoded Viral Interleukin-6**. *J Virol* 2015, **89**(15):7979-7990.
